# *Wuchereria bancrofti* infection is linked to systemic activation of CD4 and CD8 T cells

**DOI:** 10.1371/journal.pntd.0007623

**Published:** 2019-08-19

**Authors:** Inge Kroidl, Mkunde Chachage, Jonathan Mnkai, Anthony Nsojo, Myrna Berninghoff, Jaco J. Verweij, Lucas Maganga, Nyanda E. Ntinginya, Leonard Maboko, Petra Clowes, Michael Hoelscher, Elmar Saathoff, Christof Geldmacher

**Affiliations:** 1 Division of Infectious Diseases and Tropical Medicine, University Hospital, LMU Munich Germany; 2 National Institute for Medical Research (NIMR)-Mbeya Medical Research Center (MMRC), Mbeya, Tanzania; 3 German Center for Infection Research (DZIF), partner site Munich, Germany; 4 Laboratory for Medical Microbiology and Immunology, Elisabeth Tweesteden Hospital, Tilburg, The Netherlands; NIH-NIRT-ICER, INDIA

## Abstract

**Background:**

Susceptibility to HIV has been linked to systemic CD4+ T cell activation in cohorts of seronegative individuals with high HIV-exposure risk. We recently described an increased risk of HIV transmission in individuals infected with *Wuchereria bancrofti*, the causative agent for lymphatic filariasis, in a prospective cohort study. However, the reason for this phenomenon needs further investigation.

**Methodology/Principal findings:**

Two-hundred and thirty-five HIV negative adults were tested using Trop Bio ELISA for detection of *W*. *bancrofti* infection and Kato Katz urine filtration and stool based RT-PCR for detection of soil transmitted helminths and schistosomiasis. FACS analysis of the fresh peripheral whole blood was used to measure T cell activation markers (HLA-DR, CD38), differentiation markers (CD45, CD27), markers for regulatory T cells (FoxP3, CD25) and the HIV entry receptor CCR5. Frequencies of activated HLA-DR^pos^ CD4 T cells were significantly increased in subjects with *W*. *bancrofti* infection (n = 33 median: 10.71%) compared to subjects without any helminth infection (n = 42, median 6.97%, p = 0.011) or those with other helminths (*Schistosoma haematobium*, *S*. *mansoni*, *Trichuris trichiura*, *Ascaris lumbricoides*, hookworm) (n = 151, median 7.38%, p = 0.009). Similarly, a significant increase in HLA-DR^pos^CD38^pos^ CD4 T cells and effector memory cells CD4 T cells (CD45RO^pos^CD27^neg^) was observed in filarial infected participants. Multivariable analyses further confirmed a link between *W*. *bancrofti* infection and systemic activation of CD4 T cells independent of age, fever, gender or other helminth infections.

**Conclusions/Significance:**

*W*. *bancrofti* infection is linked to systemic CD4 T cell activation, which may contribute to the increased susceptibility of *W*. *bancrofti* infected individuals to HIV infection.

## Author summary

The importance of CD4 T cell activation for HIV susceptibility has been emphasized in several studies focusing on HIV transmission and prevention. Particularly, activated HLA-DR+ CD4 T cells may play a major role in HIV susceptibility. In this analysis we describe systemic activation of CD4 T cells in individuals infected with *W*. *bancrofti* the causative agent of lymphatic filariasis. This helminth disease leads to debilitating pathology in some of the individuals; however, the majority of infected persons remain asymptomatic. We recently described an increased HIV incidence in subjects infected with *W*. *bancrofti* compared to uninfected individuals from the same area. To decipher underlying reasons for this phenomenon, we measured immune activation parameters in CD4 and CD8 T cells. The increased percentage of HLADR positive and HLADR/CD38 positive CD4 T cells and also effector memory CD4 T cells that we describe here could be a possible mechanism to explain our previous findings of increased HIV incidence in individuals infected with this filarial nematode.

## Introduction

The human immunodeficiency virus (HIV) epidemic and high HIV transmission rates continue to affect large parts of the world [1]. The disproportionately high prevalence of HIV in communities of sub-Saharan Africa has led to the hypothesis that concomitant helminth infections augment the risk of HIV transmission by increasing systemic immune activation [2–5]. Helminths are parasitic worms that primarily affect the world’s poor and cause chronic infections in one-fourth of the world's population [6]. Different helminth infections can induce a diverse array of clinical symptoms and pathology. In addition to their associated morbidity, several distinct immunological changes have been described for different helminth species [7], some of which might potentially increase HIV susceptibility.

Recently, we demonstrated for the first time an increased risk of HIV acquisition in individuals infected with the filarial nematode *Wuchereria bancrofti*, the agent that causes lymphatic filariasis [8]. Our data shows that *W*. *bancrofti* is an independent risk factor for HIV acquisition even when adjusting for sexual behavior, condom use, circumcision, marital status, age and gender, among other factors.

Infections with *W*. *bancrofti* are chronic and persist over many years despite antihelminthic treatment. The majority of infected individuals remain asymptomatic; however, 10 to 30% develop filarial pathology with lymphedema or hydrocele. Different clinical outcomes are associated with distinct immunological phenotypes. The early phase of a filarial infection is characterized by proinflammatory cytokines [9], followed by anti-inflammatory cytokines such as IL-10 and TGF- beta during the chronic phase of infection in clinically asymptomatic individuals [10, 11]. In patients with chronic filarial pathology, a lack of regulatory T cells and increased Th1 and Th17 proinflammatory responses were documented [12]. Members of the family of vascular endothelial growth factors, as well as IL-1beta, TNF-alpha, and IL-6 have been implicated in lymphangiogenesis [13–15]. Individuals with lymphedema showed increased frequencies of HLA-DR^pos^ CD8 T cells [16]; however no differences in CD4 T cell maturation or expression of HLADR have yet been described for *W*. *bancrofti*-infected individuals.

Activated CD4 T cells are a major cellular reservoir for continuous HIV replication *in vivo*, as shown in several studies [17–20]. In order to address the hypothesis that the increased HIV transmission risk in *W*. *bancrofti* -infected subjects is linked to increased systemic CD4 T cell activation, we studied expression of activation (HLA-DR, CD38) and maturation (CD27, CD45RO) markers in CD4 and CD8 T cell populations, as well as T regulatory CD4 T cells and HIV entry receptor (CCR5) in relation to *W*. *bancrofti* infection status.

## Material and methods

### Ethics

This study was approved by the ethics committees of the Tanzanian National Institute for Medical Research, Mbeya Medical Research and Ethics Committee and Munich University Hospital, and was conducted according to the principles expressed in the Declaration of Helsinki. All participants recruited in the study were adults above 18 years of age who provided written informed consent before enrolment into the study.

### Study volunteers

A total of 386 adult study participants from the ‘‘Evaluating and Monitoring the Impact of New Interventions” (EMINI) general population cohort from the Mbeya region in Southwest Tanzania were enrolled into the prospective Worm-HIV-Interaction-Study (WHIS) based on their helminth and HIV infection status between October 2009 and March 2012 [21]. After excluding 136 HIV-infected study participants, 2 individuals without filariasis results, and 13 participants with invalid flow cytometry data, results from 235 HIV negative volunteers (137 women and 98 men) were analyzed. In the study area, governmental treatment of *W*. *bancrofti* started in 2009. However, none of the 235 participants reported participation in the mass drug administration program.

### Methods

This study uses the blood, urine and stool specimens that were collected from each participant at baseline. HIV status was determined using HIV 1/2 STAT-PAK (Chem-Bio Diagnostics Systems) and positive results were confirmed using ELISA (Bio-Rad) with discrepancies being resolved by Western Blot (MPD HIV Blot 2.2, MP Biomedicals). Forty ml of venous blood were drawn from each participant using anticoagulant tubes (CPDA, EDTA; BD Vacutainer). Fresh blood samples were processed within 6 hours of the blood draw at the NIMR-MMRC laboratories.

#### Diagnosis of helminth infections

*W*. *bancrofti* infection was diagnosed using an ELISA specific for circulating filarial antigen (TropBio Og4C3 serum ELISA, Townsville, Australia) as described previously [22] in bio banked cryopreserved plasma, information on the presence of microfilariae was not available. Four individuals showed *W*. *bancrofti*-associated pathology: High levels of circulating filarial antigen were measured in two individuals with hydrocele, whereas another two individuals with lymphedema of the legs had a negative ELISA result. As this phenomenon is common in filarial associated lymphedema, both participants were grouped as *W*. *bancrofti* infected. *Schistosoma haematobium* infection was diagnosed by microscopic examination of a filtered urine sample (20 ml). Fresh stool specimens were used for Kato-Katz diagnosis of intestinal nematode and *S*. *mansoni* infections [23–25]. Briefly, two Kato-Katz thick smears (41.7 mg each) were prepared from each fresh stool. Kato-Katz slides were microscopically examined for helminth eggs by experienced technicians within one hour (for hookworm eggs) and within two days (for other helminth eggs) after slide preparation. Helminth infection was defined as the presence of at least one worm egg in the examined samples.

In addition, a multiplexed RT-PCR was performed for all subjects on fecal DNA samples for simultaneous detection of *Schistosoma* species, hookworm, *Ascaris lumbricoides and Strongyloides stercoralis* [26]. For DNA amplification, 5 μL DNA extracted from 0.1 g stool specimens were used as a template in a final volume of 25 μL with PCR buffer (HotstarTaq Master Mix [5 mM MgCl_2_ and 2.5 mg bovine serum albumin]; Roche Diagnostics, Almere, The Netherlands), 2 pmol of each *A*. *lumbricoides*-specific primer, 5 pmol of each *N*. *americanus*-specific primer, 5 pmol of each *Schistosoma*-specific primer and 2.5 pmol of each *S*. *stercoralis*- specific primer. For *Necator americanus*, *A*. *lumbricoides*, *S*. *stercoralis*, and *Schistosoma*-specific double-labelled probes, 1.25 pmol were used. PCR amplification consisted of 15 minutes at 95°C followed by 50 cycles of 15 seconds at 95°C, 30 seconds at 60°C, and 30 seconds at 72°C. Amplification, detection and data analysis were performed at NIMR-MMRC in Tanzania with the Corbett Rotor-Gene 6000 Real-Time PCR System and Corbett Rotor-Gene 6000 Application Software, version 1.7. Negative and positive external control samples were included in each amplification run. The details of all primers and detection probes used in our study are described elsewhere [26–28]. Before testing of actual WHIS stool samples, molecular diagnosis of helminths at NIMR-MMRC in Tanzania was validated externally at the University of Leiden, Leiden, Netherlands, by comparing results for 19 Tanzanian fecal DNA samples. Concordance between the two laboratories was very high: 89% for *Schistosoma* species (11 of 13 positive samples) and 100% concordance for *N*. *americanus* (6 positive samples), *A*. *lumbricoides* (3 positive samples), *S*. *stercoralis* (2 positive samples) and *Ancylostoma duodenale* (no positive sample).

#### Quantification of eosinophil counts

An automated complete blood count machine (Beckman Coulter) was used for counting eosinophils. If eosinophil counts were out of range (>1000 x10^3^/μl), determination was performed using the differential blood count.

#### Characterization of markers on CD4 and CD8 T cells in fresh whole blood

Frequencies of activation (HLA-DR, CD38) and maturation (CD27, CD45RO) markers on CD4 and CD8 T cells were determined as previously described [21] in fresh, anti-coagulated whole blood. CD4^pos^ T lymphocytes were classified as “naïve” (CD27^pos^CD45RO^neg^), ‘‘central memory-like" (CD27^pos^CD45RO^pos^) and effector memory (CD27^neg^CD45RO^pos^) CD4 T cells. Gating strategies are given in the supplement. Blood samples were incubated for 10 minutes with CCR5 PECy7 (natutec, Frankfurt, Germany) followed by 30 minutes incubation using the following fluorochrome labeled monoclonal antibodies for cell surface staining (mABs): CD3 Pac Blue (Becton Dickinson; New Jersey, USA), CD4 Per-CP Cy5.5 (eBioscience, San Diego USA), CD8 V500 or CD8 Amcyan, CD25 phycoerythrin (PE)-Cy7 (eBioscience), CD27 APC-H7, CD45RO APC, HLA-DR FITC and CD38 PE (all from BD). Intracellular forkhead-box-protein P3 (FoxP3) was detected using FoxP3-Alexa Fluor 647 (ebioscience). Stained cells were then finally fixed with 2% paraformaldehyde prior to acquisition. Acquisition was performed on a FACS CANTO II (BD). Compensation was conducted with antibody capture beads (BD) stained separately with the individual antibodies used in the test samples. Flow cytometry data was analyzed using FlowJo (version 9.5.3; Tree Star Inc.).

#### Statistical analysis

GraphPad prism Version 6 was used to graphically compare single parameters between groups. Since most outcomes were not normally distributed, we used the Kruskall Wallis test to compare groups overall and the Mann-Whitney test for comparisons between two groups. To assess the influence of filariasis infection on different markers of immune status, we adjusted for potential confounders using Stata version 14.1 statistics software. We constructed mixed linear regression models with area of residence as a random effect for both uni-variable analysis and multi-variable analysis and adjusted for potential confounders (age, gender, infections with different helminths and the presence of fever in the last 24 hours).

## Results

**Fig 1:**
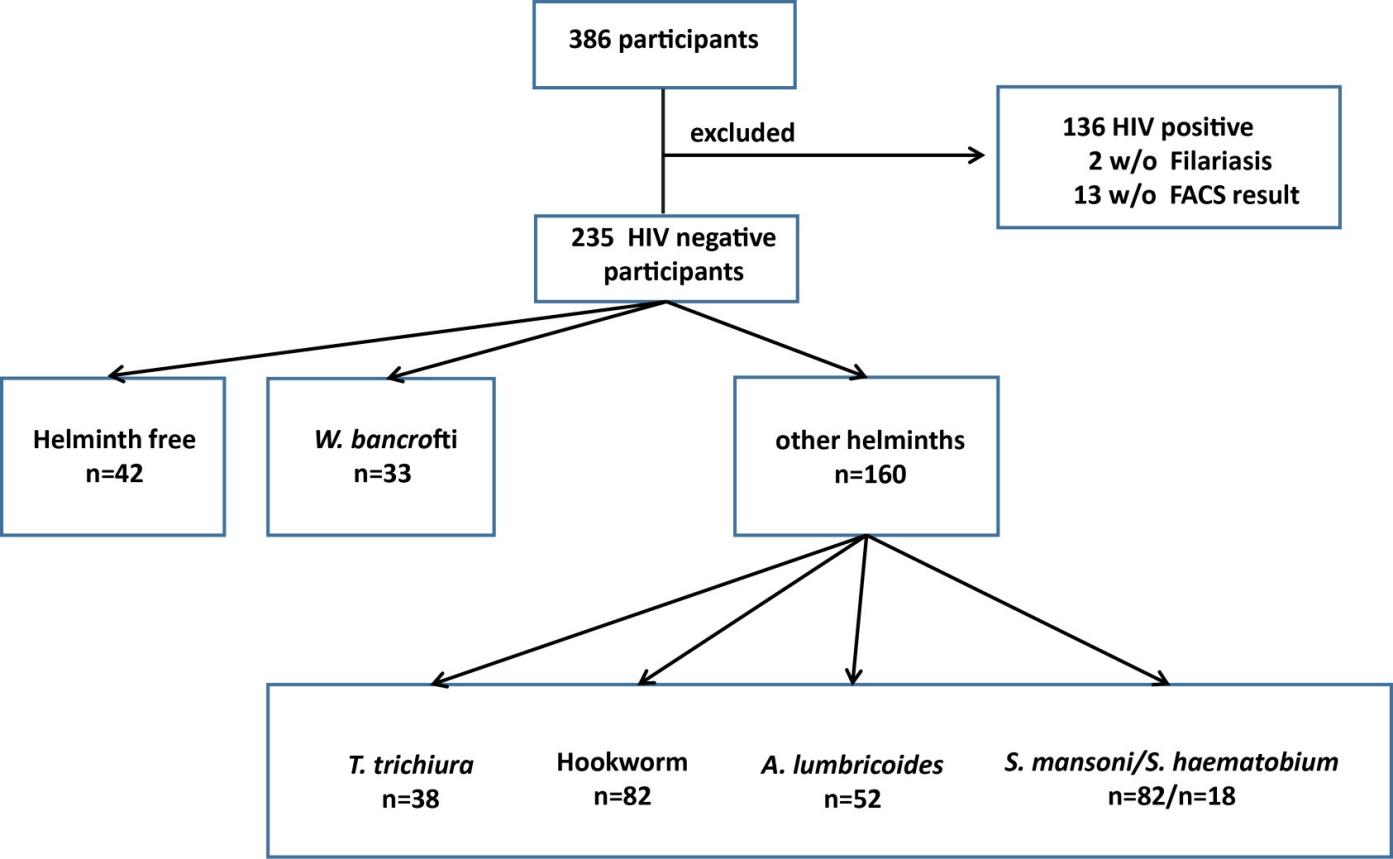
Participants and their grouping according to HIV and helminth infection status. For the analysis only HIV negative individuals with valid data for flow cytometry and *W*. *bancrofti* were included. The participants were separated into three different sub groups-.

Among the 235 HIV negative study subjects, 42 (17.9%) were helminth negative and 193 (82.1%) were helminth positive (Fig 1). *W*. *bancrofti* infection was detected in 33 subjects (16.1%). Other helminth infections included *S*. *mansoni* (82 cases, 42.5%), *S*. *haematobium* (18 cases, 9.3%), *N*. *americanus* (hookworm) (82 cases, 42.5%), *A*. *lumbricoides* (52 cases, 26.9%) and *Trichuris trichiura* (38 cases, 19.7%) (Fig 1). Of the 193 individuals with diagnosed helminth infection, 104 (53.9%) were infected with one helminth species, 66 (34.2%) with two species, and 23 (11.9%) with 3 or more helminth species.

**Fig 2:**
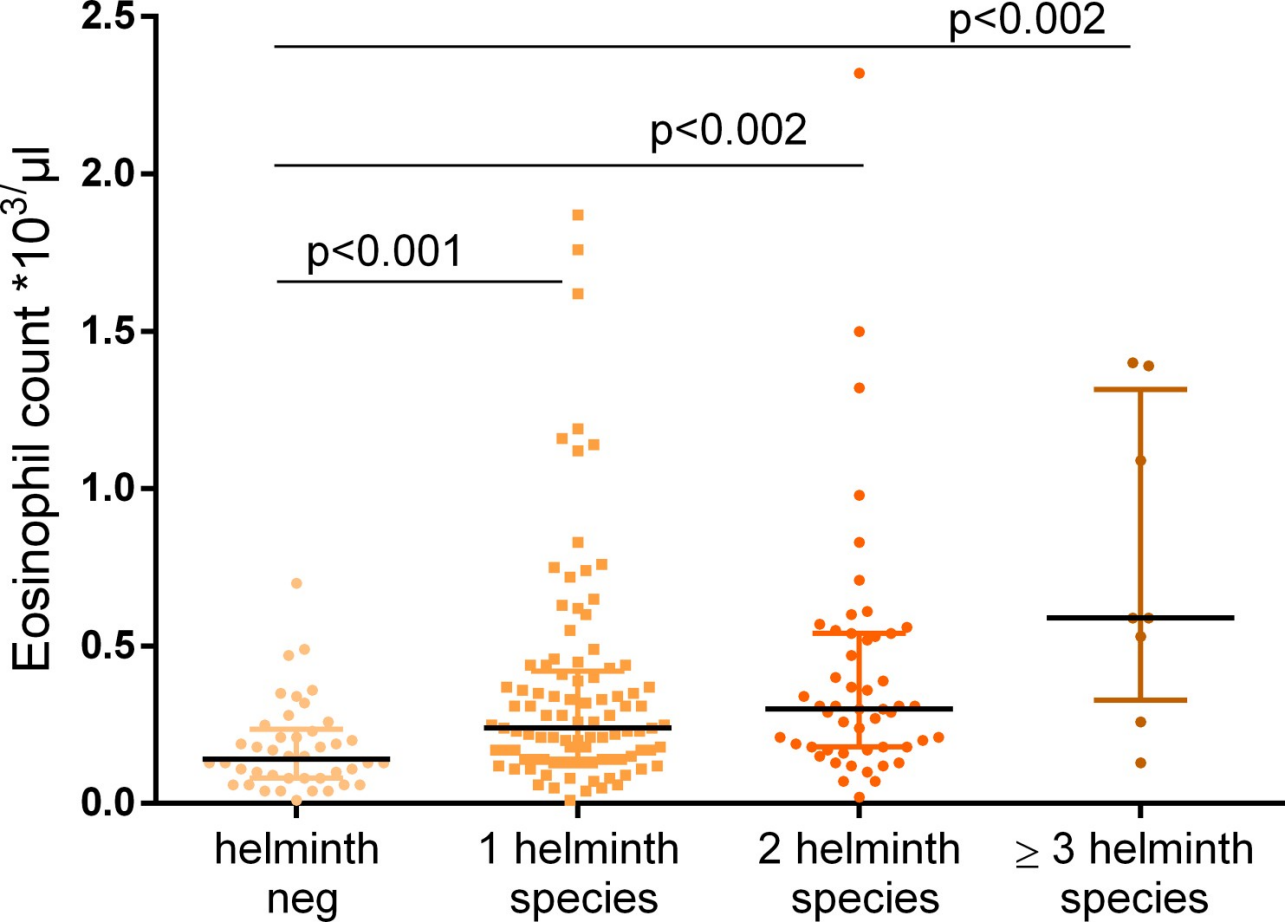
Eosinophil counts and polyparasitism. Eosinophil counts were available for 37 study participants without helminth infection, 87 with one helminth species, 46 with two species, and 7 with three or more helminth species. Increasing numbers of eosinophils were seen with polyparasitism. *Kruskal-Wallis* testing showed significant differences between groups overall (p = 0.0001) as well as *Mann-Whitney* testing to compare the groups separately.

Almost all *W*. *bancrofti*-infected subjects (32 of 33) were also infected with other helminths (Table 1) impeding an evaluation of only monoinfected individuals. A mean of 2.3 helminths species were detected in *W*. *bancrofti*-infected individuals. The composition of the “additional” species in *W*. *bancrofti*-infected individuals was almost identical to that in the group harboring other helminth species (Table 1). Therefore we compared the effect of *W*. *bancrofti* on T cell activation not only with the group of helminth free individuals, but in addition with the “background” infection of the combined helminth group to distinguish the effect of *W*. *bancrofti* from an overall helminth influence on immune activation. The median age in the three groups ranged from 31 to 37 years and the percentage of males was between 39% and 48%. All three groups included less than 10% of individuals with self-reported fever during the last 24 hours (Table 1). As expected, there was a difference in eosinophil count between helminth free individuals and participants with one of the diagnosed helminth species. We found a median number of 130x10^3^/μl eosinophils in helminth free individuals compared to 400x10^3^/μl in *W*. *bancrofti-*infected individuals and 260x10^3^/μl in participants harboring other helminths. We also found that eosinophil counts increased in parallel with the number of helminth infections (Fig 2).

**Table 1:** Participant characteristics by helminth status. The group of *W*. *bancrofti* infected individuals was compared to helminth–free individuals and a group of participant infected with other helminth species. For each group the median age, gender distribution, pathology, the mean number of different helminth species, and the percentage of each type of helminth in the respective group, median eosinophil count and percentage of individuals with fever is given.

	Helminth negative individuals	*W*. *bancrofti—*infected individuals	Combined group of individuals infected with other helminths
**N**	42	33	160
**Age** **median** **(range)**	37.2 (18.4 to 76.0)	31.6 (19.1 to 54.0)	31.4 (18.0 to 62.4)
**Male gender,** **N (%)**	19 (45%)	16 (48%)	63 (39%)
**Filarial pathology** **Hydrocele** **Lymphedema**	0 0	2 (6%) 2 (6%)	0 0
**mean number of different helminth species**	0	2.3	1.4
**Additional helminths** **None** ***Schistosoma* spec.** **Hookworm** ***A*. *lumbricoides*** ***T*. *trichiura***	42 (100%) 0 0 0 0	1 (3%) 14 (42%) 12 (36%) 9 (27%) 13 (39%)	0 82 (51%) 70 (44%) 43 (27%) 25 (16%)
Eosinophil count *10^**3**^**/μl** **median** **(IQR)**	130 (80 to 230)	400 (260 to 490)	260 (150 to 455)
**Fever****, N (%)**	4 (9.5%)	2 (6.1%)	14 (8.8%)

### Increased systemic T cell activation of individuals infected with *W*. *bancrofti*

**Fig 3:**
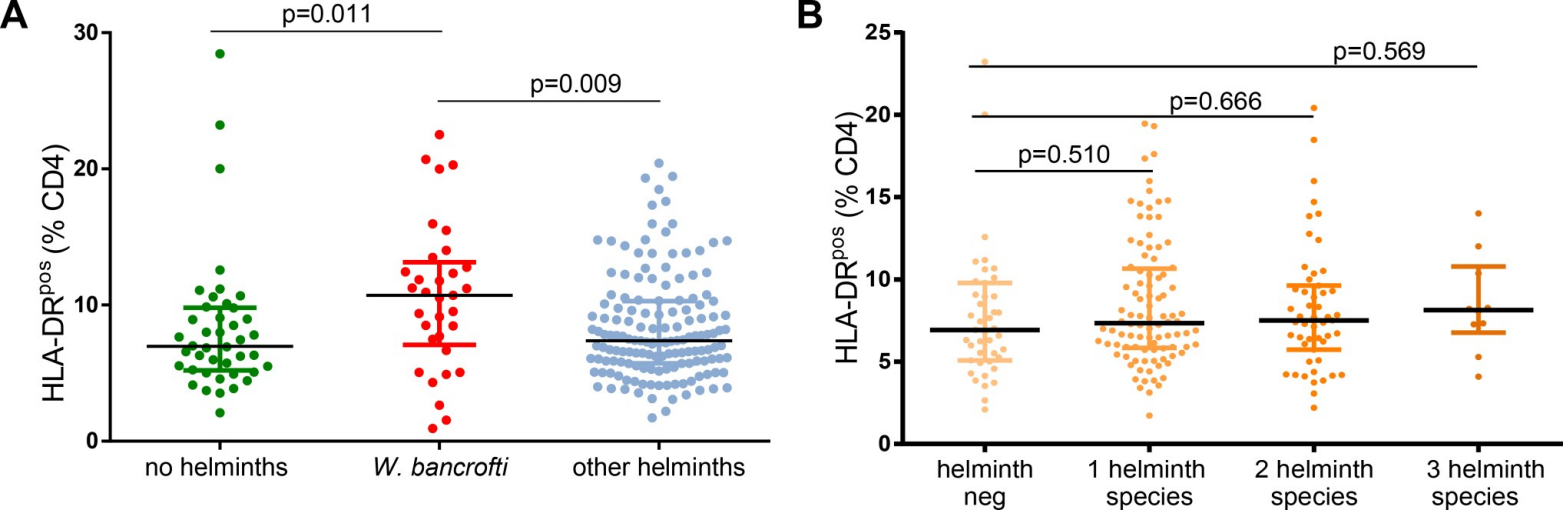
**A: Expression of HLADR on CD4 T cells in relation to the helminth infection status.** The percentage of HLADR expressing CD4 T cells is shown for the three categories of participants. *Kruskal-Wallis* testing showed significant difference between groups overall (p = 0.0168). *W*. *bancrofti-*infected individuals had significantly more HLADR expressing CD4 T cells compared to helminth free individuals or study participants infected with other helminths as shown by *Mann-Whitney* test. **B: Association of polyparasitism with activation status.** Number of eosinophils is shown for participants with increasing numbers of helminth species. *Kruskal-Wallis* testing showed no significant difference between groups (p = 0.917) as well as *Mann-Whitney* test.

**Fig 4:**
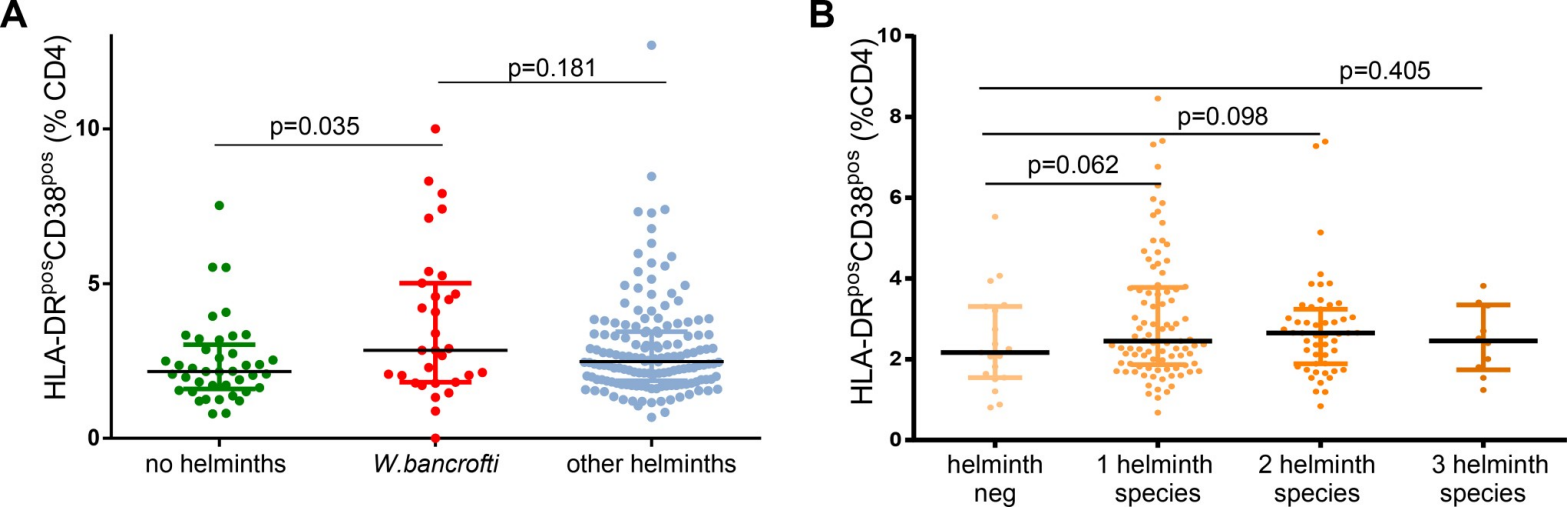
**A: Association of helminth status with the frequency of HLA-DR**^**pos**^ **CD38**^**pos**^ **CD4 T cells.** The percentage of HLA-DR^+^ CD38^+^ expressing CD4 T cells is shown for the three categories of participants. *Kruskal-Wallis* testing showed significant difference between groups overall (p = 0.044). A significant difference was seen between the subgroup of individuals without helminths and the *W*. *bancrofti*- infected participants (using *Mann-Whitney* testing). **B: Association of polyparasitism with the frequency of HLA-DR**^**+**^ **CD38**^**+**^ **CD4 T cells**. The percentage of HLA-DR^+^ CD38^+^ expressing CD4 T cells is shown for participants with increasing numbers of helminth species. *Kruskal-Wallis* testing showed no significant difference between groups (p = 0.262), as did the Mann-Whitney.

Fresh whole blood was used for flow cytometric assessment of activation and maturation markers on T cells. Frequencies of HLA-DR^pos^ CD4 T cells were significantly increased in subjects with *W*. *bancrofti* infection (n = 33 median: 10.71%) compared to subjects either without any helminth infection (n = 42, median 6.97%, p = 0.016 Mann-Whitney test, univariable analysis, Fig 3A) or the combined group of individuals harboring other helminths (*S*. *haematobium*, *S*. *mansoni*, *T*. *trichiura*, *A*. *lumbricoides*, hookworms) (n = 151 median 7.38%, p = 0.009). Polyparasitism was not significantly associated with increased frequencies of HLA-DR^pos^ CD4 T cells (Fig 3B). *W*. *bancrofti* infection was associated with increased frequencies of HLA-DR^pos^CD38^pos^ CD4 T cells (n = 31) compared to helminth negative subjects (n = 41median 2.84% versus 2.16%, p = 0.035, Fig 4A). Again, polyparasitism was not significantly associated with increased frequencies of HLA-DR^pos^CD38^pos^ CD4 T cells (Fig 4B).

To adjust the above associations of filariasis infection with immune activation for potentially confounding factors, we estimated multivariable mixed effects models including age, gender, fever and the other helminth infections (*T*. *trichiura*, *A*. *lumbricoides*, *S*. *mansoni*, *S*. *haematobium* and hookworm) as fixed effects and study site as a random effect in linear regression models. Univariable analysis had demonstrated an influence of these factors on our outcome parameters (Tables 2 and S1–S8). The significant association of *W*. *bancrofti* infection with HLA-DR^pos^ CD4 T cell counts was confirmed in the multivariable analysis (Table 2).

**Table 2:** Association of helminth infections with percent of HLA-DR^pos^ CD4 T cells. Uni- and multi-variable mixed-effects linear regression results, with random effect for residence in Kyela site, multivariable results adjusted for all variables shown.

** **			univariable			multivariable		
Covariate	N	Mean	Coef.	95% CI	p-value	Coef.	95% CI	p-value
**Age**								
**(per year)**	-	-	0,13	(0.08 to 0.18)	0.0000	0,14	(0.09 to 0.18)	0.0000
**Sex**								
female*	131	8,78	0,00	-	-	0,00	-	-
**male**	95	8,45	-0,56	(-1.71 to 0.58)	0.3332	0,15	(-0.92 to 1.21)	0.7887
**Current fever**								
no*	200	8,50	0,00	-	-	0,00	-	-
**yes**	20	10,39	1,99	(0.04 to 3.95)	0.0460	1,36	(-0.45 to 3.18)	0.1412
**no data**	6	7,39	-0,48	(-3.96 to 3.00)	0.7860	-0,13	(-3.35 to 3.09)	0.9359
***W*. *bancrofti***								
neg. *	193	8,31	0,00	-	-	0,00	-	-
**pos.**	33	10,59	1,83	(0.14 to 3.52)	0.0337	1,58	(0.08 to 3.09)	0.0392
**Hookworm**								
neg. *	147	8,83	0,00	-	-	0,00	-	-
**pos.**	79	8,29	-0,62	(-1.79 to 0.55)	0.3019	-0,74	(-1.83 to 0.35)	0.1827
***A*. *lumbricoides***								
neg. *	177	8,25	0,00	-	-	0,00	-	-
**pos.**	49	10,05	1,49	(0.11 to 2.86)	0.0337	2,12	(0.80 to 3.43)	0.0016
***T*. *trichiura***								
neg. *	189	8,34	0,00	-	-	0,00	-	-
**pos.**	37	10,15	1,15	(-0.53 to 2.83)	0.1807	1,82	(0.34 to 3.31)	0.0162
***S*. *mansoni***								
neg. *	145	8,64	0,00	-	-	0,00	-	-
**pos.**	81	8,63	-0,09	(-1.26 to 1.08)	0.8839	1,46	(0.28 to 2.65)	0.0155
***S*. *haematobium***								
neg. *	209	8,67	0,00	-	-	0,00	-	-
**pos.**	17	8,22	-0,48	(-2.60 to 1.64)	0.6573	0,71	(-1.26 to 2.67)	0.4818

N = number of observations; Mean = mean outcome; Coef. = coefficient; 95% CI = 95% confidence interval

* reference stratum

Table 2 shows HLA-DR^pos^ CD4 T cell counts of all tested helminths. Some other helminth species, for example *A*. *lumbricoides and T*. *trichiura also* show elevated HLA-DR^pos^ CD4 T cells in uni- and multivariable analysis. This was described and discussed in a previous manuscript about this cohort [21] and is not repeated in this manuscript. In contrast, other helminth species, for example hookworms seem to have no impact on immune activation marker.

Similar uni- and multivariable analyses were performed for HLA-DR^pos^ CD8 T cell counts as well as for HLA-DR^pos^ CD38^pos^ CD4 and HLA-DR^pos^ CD38^pos^ CD8 T cell counts (Tables 3 and S1, S2, S3).

**Table 3:** Associations of filariasis infection with different markers of immune activation. Separate uni- and multi-variable mixed-effects linear regression models for each marker with random effect for residence at Kyela site. Differences in number of observations are due to missing outcome data.

				Univariable*			Multivariable**		
Outcome		N	Mean	Coef.	95% CI	p-value	Coef.	95% CI	p-value
**% HLA-DR**^**pos**^ **cells of all CD4 T cells**				** **					
	*W*. *bancrofti*								
	neg. *	193	8,31	0,00	-	-	0,00	-	-
	pos.	33	10,59	1,83	(0.14 to 3.52)	0.033	1,58	(0.08 to 3.09)	0.039
**% HLA-DR**^**pos**^**CD38**^**pos**^ **cells of all CD4 T cells**				** **					
	*W*. *bancrofti*								
	neg. *	189	2,82	0,00	-	-	0,00	-	-
	pos.	31	3,69	0,86	(0.21 to 1.52)	0.009	0,84	(0.19 to 1.50)	0.011
**% HLA-DR**^**pos**^ **cells of all CD8 T cells**				** **					
	*W*. *bancrofti*								
	neg. *	192	20,8	0,00	-	-	0,00	-	-
	pos.	33	29,5	1,69	(-3.18 to 6.56)	0.496	0,47	(-3.96 to 4.89)	0.836
**% HLA-DR**^**pos**^**CD38**^**pos**^ **cells of all CD8 T cells**				** **					
	*W*. *bancrofti*								
	neg. *	189	7,69	0,00	-	-	0,00	-	-
	pos.	32	13,42	3,49	(0.94 to 6.04)	0.007	3,45	(0.91 to 5.98)	0.007
**% CD27**^**neg**^**CD45RO**^**pos**^ **cells of all CD4 T cells**				** **					
	*W*. *bancrofti*								
	neg. *	189	20,04	0,00	-	-	0,00	-	-
	pos.	31	24,10	4,06	(0.99 to 7.13)	0.009	3,16	(0.17 to 6.15)	0.038
**% CD25**^**high**^**FOXP3**^**pos**^ **cells of all CD4 T cells**				** **					
	*W*. *bancrofti*								
	neg. *	178	2,258	0,00	-	-	0,00	-	-
	pos.	30	2,453	0,13	(-0.30 to 0.57)	0.549	0,03	(-0.40 to 0.45)	0.898
**% CCR5**^**pos**^ **cells of all CD4 T cells**				** **					
	*W*. *bancrofti*								
	neg. *	170	24,06	0,00	-	-	0,00	-	-
	pos.	30	25,15	1,09	(-2.29 to 4.47)	0.528	1,45	(-2.03 to 4.93)	0.414
**% CCR5**^**pos**^ **cells of regulatory CD4 T cells**				** **					
	*W*. *bancrofti*								
	neg. *	170	54,47	0,00	-	-	0,00	-	-
	pos.	30	55,80	1,34	(-3.99 to 6.66)	0.622	2,55	(-2.89 to 8.00)	0.358
**mean fluorescence intensity of CCR5 on memory CD4 T cells**									
	*W*. *bancrofti*								
	neg. *	185	799	0,00	-	-	0,00	-	-
** **	pos.	31	699	-100,43	(-244.95 to 44.10)	0.173	-65,07	(-214.22 to 84.09)	0.392

N = number of observations; Mean = mean outcome; Coef. = coefficient; 95% CI = 95% confidence interval

* unadjusted, but with random effect for Kyela site

** with random effect for Kyela site and adjusted for age, sex, current fever, and for hookworm, *A*. *lumbricoides*, *T*. *trichiura*, *S*. *mansoni & haematobium* infection. Full models showing all co-variates are contained in the supplement.

The significant increase in activated T cells in *W*. *bancrofti-*infected individuals was confirmed for the HLA-DR^pos^ CD38^pos^ CD4 and CD8 T cells. Frequencies of HLA-DR^pos^ CD8 T cell were increased in subjects with *W*. *bancrofti* (mean 29.5%) infection compared to subjects without filarial infection (mean 20.8%). However, this difference was not significant after adjusting for study site and other confounding factors in the multivariable analysis (Tables 3 and S2). To give an overview of the different parameters associated with immune activation, which are influenced by infection with *W*. *bancrofti*, we summarized the results in Table 3 whereas a complete multivariable analysis showing the influence of other factors are given in the supplementary Tables S1–S8.

**Fig 5:**
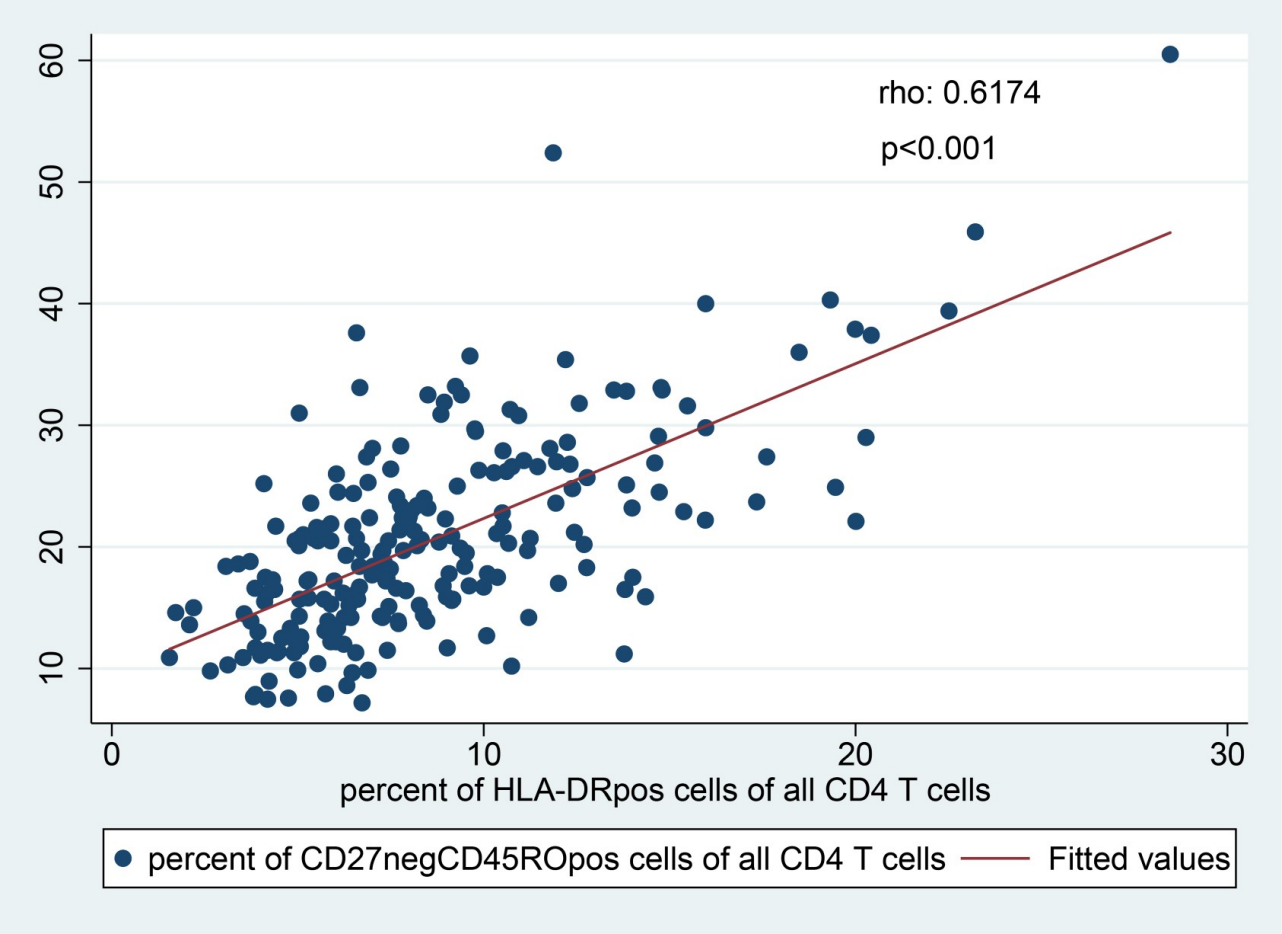
Correlation of effector memory cells (CD27^neg^CD45RO^pos^ CD4 T cells) and activation status (HLADR^pos^ CD4 T cells). A positive correlation was found (Spearman’s rho = 0.617, p<0.001).

Apart from T cell activation, we also studied the memory phenotype of CD4 T cells using CD45RO and CD27 as markers for naïve (CD27^pos^CD45RO^neg^), ‘‘central memory-like" (CD27^pos^CD45RO^pos^) and effector memory (CD27^neg^CD45RO^pos^) CD4 T cells. Total memory CD4 T cells were defined as the sum of central memory (CD27^pos^CD45RO^pos^), effector memory (CD27^neg^CD45RO^pos^) and “terminally differentiated” (CD27^neg^CD45RO^neg^) CD4 T cells. *W*. *bancrofti* infection was associated with significantly increased frequencies of effector memory (CD27^neg^CD45RO^pos^) CD4 T cells, in univariable (mean 24.1% versus 20.0%, p = 0.0096) as well as multivariable analysis (Tables 3 and S5). This was not observed for other analyzed helminth species. Importantly, the effector memory CD4 T cell frequencies were positively correlated with the activation status (Spearman’s rho = 0.617, p<0.001, Fig 5).

Regulatory CD4 T cells, defined as CD25^high^FOXP3^pos^ CD4 T cells were found in similar percentages in all study groups (Tables 3 and S4) with the exception of *T*. *trichiura*-infected individuals, who exhibited increased regulatory CD4 T cells compared to *T*. *trichiura*-uninfected individuals. Addressing the expression of HIV entry receptor CCR5, no difference was found regarding the percentage of CCR5 positive CD4 T cells, CCR5 positive regulatory CD4 T cells, or the mean fluorescence intensity of CCR5 on memory CD4 T cells between the different helminth infections (Tables 3, S6, S7 and S8).

## Discussion

The importance of CD4 T cell activation for HIV susceptibility has been emphasized in several studies that focused on HIV exposed but seronegative individuals [18–20, 29–31]. Activated CD4 T cells are a major cellular reservoir for continuous HIV replication *in vivo*. Recently, a study embedded into a phase 2B HIV vaccine trial and conducted in a high-risk setting examined a variety of different immune cell populations and phenotypes in peripheral blood in relation to HIV acquisition. The study showed that of all studied cell subsets, the frequency of activated HLA-DR^pos^ CD4 T cells discriminated best between subjects who later HIV seroconverted and age-, ethnicity- and risk-matched controls who did not seroconvert, regardless of intervention arm [17].

To address potential mechanisms underlying the *W*. *bancrofti*-associated risk increase in HIV acquisition, which was recently described by our group [8], an evaluation of different markers for maturation, immune activation as well as CCR5 expression was therefore performed. We found significantly higher frequencies of HLA-DR^pos^ and HLA-DR^pos^/CD38^pos^ as well as ‘‘effector-like” memory (CD27^neg^CD45RO^pos^) CD4 T cells in participants infected with *W*. *bancrofti* compared to individuals with other helminth infections or without helminth infection.

Univariable analysis results were supported by multivariable analyses, in which potential confounders were addressed. In our previous publications about the EMINI cohort, we had described different age and gender distributions for the detected helminth species [22–25]. We therefore included age, gender, and additionally fever (during the last 24 hours as proxy for an acute illness) into the model. In addition, we included the infection status of other helminths to adjust for potential confounding, since we had previously described a positive association of *Trichuris trichiura* and *Ascaris lumbricoides* with the expression of markers for systemic T cell activation [21]. The association between increased frequencies of activated HLA-DR^pos^ and HLA-DR^pos^/CD38^pos^ and effector memory (CD27^neg^CD45RO^pos^) CD4 T cells in peripheral blood and *W*. *bancrofti* infection was independent of other host and environmental factors, suggesting a causal link between these two factors.

The participants studied here had been selected from the large general population cohort (EMINI) based on their helminth infection status. These participants were selected in 9 different study sites with distinct geographical conditions. Participants with *W*. *bancrofti* and *T*. *trichiura* infection were almost exclusively found in Kyela site close to Lake Nyasa, whereas hookworm, *A*. *lumbricoides* and *Schistosoma* infections were found in several study sites. We therefore included residence in Kyela site as a random effect into our multivariable model. The positive association of *W*. *bancrofti* with HLA-DR^pos^ and HLA-DR^pos^/CD38^pos^ as well as ‘‘effector-like” memory (CD27^neg^CD45RO^pos^) CD4 T cells was confirmed in the mixed linear regression models with area of residence as a random effect. In addition we measured increased percentage of HLA-DR^pos^/CD38^pos^ CD8 T cells in *W*. *bancrofti*-infected individuals, however, there is no evidence that CD8 T cell may contribute to increased HIV susceptibility.

Our study has some limitations that we tried to address during the analysis. Because our cohort did not include *W*. *bancrofti* mono-infected individuals, we compared not only to helminth negative individuals, but also to individuals with other helminth infections. The participants in the study group with “other helminths” were infected with similar species in an almost identical composition compared to the *W*. *bancrofti* group, providing an optimal “background” to study the effect of an additional helminth species (Figs 3A and 4A). In addition, we showed that polyparasitism had no influence on immune activation (Figs 3B and 4B). This approach seems appropriate, as we might have missed more parasites, like *Onchocerca volvulus* and *Mansonella perstans*, *two other tissue nematodes*. Increasing information about the modulation of the immune response to other pathogens in co-infected individuals is available [32–36]. However, the study area is not known to be endemic for *O*. *volvulus* and we did not measure *M*. *perstans*.

In a previous publication [21], we described differences between helminth species regarding their association with T cell activation. With adjusting for other helminths in our multivariate analysis, we clearly showed that the described CD4 T cell activation in *W*. *bancrofti-* (but also in *A*. *lumbricoides and T*. *trichiura)* -infected individuals was independent of concomitant infection with other helminths. *W*. *bancrofti* infection was diagnosed by retrospective testing of bio-banked samples using an ELISA specific for circulating filarial antigen in plasma (TropBio Og4C3 ELISA, Townsville, Australia), which was an identical diagnostic approach to what was used in our previous study that showed significantly increased HIV acquisition risk in *W*. *bancrofti-*infected subjects [8]. It would have been interesting to characterize *W*. *bancrofti* infection in more detail, such as investigating the presence of microfilariae (the offspring of the adult worms, responsible for *W*. *bancrofti* transmission from humans to mosquitos), which requires the nocturnal collection of blood. Unfortunately, we do not have information about microfilariae for our study participants.

Because the clinical presentation of *W*. *bancrofti* infection shows such variety, the immunological background of these features has been studied extensively: Hyporesponsiveness was reported from in vitro studies of PBMC cultures of *W*. *bancrofti*-infected individuals [37, 38]. Treatment of the filarial parasites resulted in recovery of antigen-specific responses supporting the direct causal relationship [39]. It was discovered that the hyporesponsiveness, which was described initially, was typical for the filarial infected but asymptomatic individuals, whereas in lymphedema patients Th1 and Th17 responses mediated the pathology [12]. However, the number of participants with pathology in our cohort was too small to describe differences among sub groups. Infected individuals, as measured by the presence of circulating filarial antigen, who do not have microfilariae in the night blood, display a distinct immunological profile, characterized by stronger filarial-specific interleukin-5, IL-10 and TNF-alpha responses, compared to microfilaremic individuals [40]. Regulatory T cells, which are responsible for tolerance to self-antigens, show stronger activity in microfilaremic, compared to amicrofilaremic individuals [41–44]. In this context, it is possible that also HLA-DR^pos^ and HLA-DR^pos^/CD38^pos^ CD4 T cell frequencies, and also expression of HIV entry receptors, may differ in microfilaremic, amicrofilaremic or symptomatic filarial infected patients. Further studies with a better characterization of participants are needed.

An increase of systemically activated CD4 T cells is likely to support early HIV dissemination because they are cellular targets for HIV replication [45, 46]. However, it is unknown whether increased activation of circulating T cells in peripheral blood is also linked to increased activation of CD4 T cells at the mucosal site of viral transmission. In our study, the frequency of HLA-DR^pos^ CD4 T cells strongly correlates with the frequency of effector memory CD4 T cells and both subsets are significantly increased *W*. *bancrofti* infected subjects. We have previously shown in the same cohort that both CD27 negative effector memory CD4 T cells and HLA-DR+ memory CD4 T cells express significantly higher levels of the HIV co-receptor CCR5 compared to CD27+ central memory or naive CD4 T cells [21], which may facilitate better HIV entry into these specific cell subsets. These effector memory CD4 T cells may migrate into tissues [47], such as the mucosal surfaces of the reproductive tract. Thus, future studies need to study HIV co-receptor expression and T cell activation in the reproductive tract in relation to *W*. *bancrofti* infection.

**In summary,** our data clearly show that *W*. *bancrofti* infection is linked to systemic CD4 T cell activation, as determined by HLA-DR expression, providing a possible mechanism for the increased HIV-susceptibility observed in *W*. *bancrofti-*infected individuals [8]. Whether treatment of *W*. *bancrofti* could decrease immune activation and whether this in turn could reduce HIV transmission in areas where lymphatic filariasis is prevalent needs to be examined in future studies.

## Supporting information

S1 Fig Gating strategy for Lymphocytes, CD3^pos^ T cells, CD4^pos^ T cells, memory and naïve, CD4^pos^ T cells, HLADR^pos^CD38^pos^ CD4 T cells, and percentage of CCR5 positive CD3 ^pos^ CD4 ^pos^ T lymphocytes. (DOCX)

S1 Table Association of various factors with percent of HLA-DR^pos^CD38^pos^ cells of all CD4 T cells (N = 220; Mean = 2.95). Uni- and multi-variable mixed-effects linear regression results, with random effect for residence in Kyela site, multivariable models additionally adjusted for age, gender and fever during last 24 hours and different helminth infections.
(DOCX)

S2 Table Association of various factors with percent of HLA-DR^pos^ cells of all CD8 T cells (N = 225; Mean = 22.1). Uni- and multi-variable mixed-effects linear regression results, with random effect for residence in Kyela site, multivariable models additionally adjusted for age, gender and fever during last 24 hours and different helminth infections.
(DOCX)

S3 Table Association of various factors with percent of HLA-DR^pos^CD38^pos^ cells of all CD8 T cells (N = 221; Mean = 8.52). Uni- and multi-variable mixed-effects linear regression results, with random effect for residence in Kyela site, multivariable models additionally adjusted for age, gender and fever during last 24 hours and different helminth infections.
(DOCX)

S4 Table Association of various factors with percent of “effector memory” CD27^neg^CD45RO^pos^ cells of all CD4 T cells (N = 220; Mean = 20.62). Uni- and multi-variable mixed-effects linear regression results, with random effect for residence in Kyela site, multivariable models additionally adjusted for age, gender and fever during last 24 hours and different helminth infections.
(DOCX)

S5 Table Association of various factors with percent of CD25^high^FOXP3^pos^ cells of all CD4 T cells (N = 208; Mean = 2.287). Uni- and multi-variable mixed-effects linear regression results, with random effect for residence in Kyela site, multivariable models additionally adjusted for age, gender and fever during last 24 hours and different helminth infections.
(DOCX)

S6 Table Association of various factors with percent of CCR5^pos^ cells of all CD4 T cells (N = 200; Mean = 24.22). Uni- and multi-variable mixed-effects linear regression results, with random effect for residence in Kyela site, multivariable models additionally adjusted for age, gender and fever during last 24 hours and different helminth infections.
(DOCX)

S7 Table Association of various factors with percentage of CCR5^pos^ of all regulatory CD4 T cells (N = 200; Mean = 54.67). Uni- and multi-variable mixed-effects linear regression results, with random effect for residence in Kyela site, multivariable models additionally adjusted for age, gender and fever during last 24 hours and different helminth infections.
(DOCX)

S8 Table Association of various factors with mean fluorescence intensity of CCR5 on memory CD4 T cells (N = 216; Mean = 785). Uni- and multi-variable mixed-effects linear regression results, with random effect for residence in Kyela site, multivariable models additionally adjusted for age, gender and fever during last 24 hours and different helminth infections.
(DOCX)
