# Supplementary material for: Wuchereria bancrofti infection is linked to systemic activation of CD4 and CD8 T cells
Source: PLoS Negl Trop Dis. 2019 Aug 19;13(8):e0007623. doi: 10.1371/journal.pntd.0007623 (PMC6736309; doi:10.1371/journal.pntd.0007623)
Supplement: S1 Fig — (DOCX) [file pntd.0007623.s001.docx]

**Supplement: manuscript „*Wuchereria bancrofti* infection is linked to systemic activation of CD4 and CD8 T cells" (#PNTD-D-19-00165 R1).**

**S1 Figure: Gating strategy**

**Measurements were done on CD3 ^pos^ CD4 ^pos^ T lymphocytes**

Lymphocytes CD3^pos^ T cells CD4^pos^ T cells

**Maturation status of CD3 ^pos^ CD4 ^pos^ T lymphocytes**

The memory phenotype of CD4 T cells was defined using CD45RO and CD27 as markers for naïve (CD27^pos^CD45RO^neg^ , left upper corner), ‘‘central memory-like" (CD27^pos^CD45RO^pos^, right upper corner) and effector memory (CD27^neg^CD45RO^pos^CD4 T cells right lower corner).

**Activation status of CD3 ^pos^ CD4 ^pos^ T lymphocytes**

The cut-off line for immune activation marker was chosen after performing FMO controls (Fluorescence minus one) and corrected after visualization of the result: HLADR^pos^CD38^pos^ CD4 T cells are in the upper right corner (8.75%). HLAD^pos^ CD4 T cells are calculated adding the upper and lower right corner.

**Percentage of CCR5 positive CD3 ^pos^ CD4 ^pos^ T lymphocytes**

For measuring the percentage of CCR5 positive CD4 T cells naïve cells were compared with all CD4 T cells. As naïve T cells should not express CCR5, the cut off was placed accordingly.


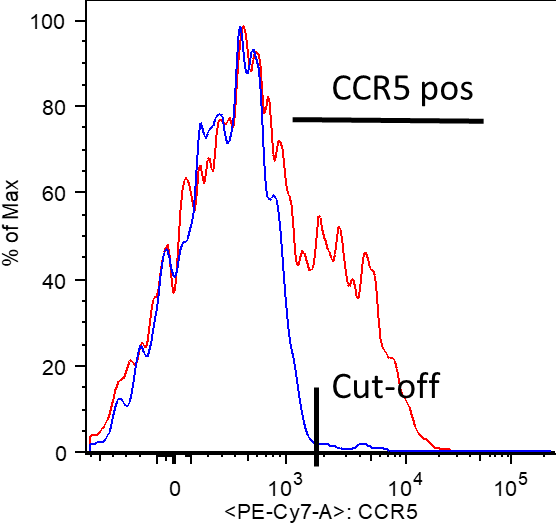


Naïve (CD27^pos^CD45RO^neg^) CD4 T cells in blue, CCR5 measurement in red
