# Supplementary material for: Wuchereria bancrofti infection is linked to systemic activation of CD4 and CD8 T cells
Source: PLoS Negl Trop Dis. 2019 Aug 19;13(8):e0007623. doi: 10.1371/journal.pntd.0007623 (PMC6736309; doi:10.1371/journal.pntd.0007623)
Supplement: S1 Table — Uni- and multi-variable mixed-effects linear regression results, with random effect for residence in Kyela site, multivariable models additionally adjusted for age, gender and fever during last 24 hours and different helminth infections. (DOCX) [file pntd.0007623.s002.docx]

**S1 Table: Association of various factors with percent of HLA-DR^pos^CD38^pos^ cells of all CD4 T cells**

|  |  |  | **univariable** | | | **multivariable** | | |
| --- | --- | --- | --- | --- | --- | --- | --- | --- |
| **Covariate** | **N** | **Mean** | **Coef.** | **95% CI** | **p-value** | **Coef.** | **95% CI** | **p-value** |
|  |  |  |  |  |  |  |  |  |
| **Age** |  |  |  |  |  |  |  |  |
| **(per year)** | - | - | 0,01 | (-0.01 to 0.03) | 0.1826 | 0,01 | (-0.01 to 0.03) | 0.1619 |
|  |  |  |  |  |  |  |  |  |
| **Sex** |  |  |  |  |  |  |  |  |
| **female*** | 126 | 3,04 | 0,00 | - | - | 0,00 | - | - |
| **male** | 94 | 2,82 | -0,28 | (-0.74 to 0.18) | 0.2386 | -0,10 | (-0.55 to 0.36) | 0.6731 |
|  |  |  |  |  |  |  |  |  |
| **Current fever** |  |  |  |  |  |  |  |  |
| **no*** | 194 | 2,90 | 0,00 | - | - | 0,00 | - | - |
| **yes** | 20 | 3,64 | 0,76 | (-0.02 to 1.55) | 0.0571 | 0,59 | (-0.17 to 1.36) | 0.1273 |
| **no data** | 6 | 1,97 | -0,79 | (-2.19 to 0.60) | 0.2654 | -0,71 | (-2.06 to 0.65) | 0.3054 |
|  |  |  |  |  |  |  |  |  |
| ***W. bancrofti*** |  |  |  |  |  |  |  |  |
| **neg.*** | 189 | 2,82 | 0,00 | - | - | 0,00 | - | - |
| **pos.** | 31 | 3,69 | 0,86 | (0.21 to 1.52) | 0.0092 | 0,84 | (0.19 to 1.50) | 0.0117 |
|  |  |  |  |  |  |  |  |  |
| **Hookworm** |  |  |  |  |  |  |  |  |
| **neg.*** | 144 | 3,06 | 0,00 | - | - | 0,00 | - | - |
| **pos.** | 76 | 2,74 | -0,33 | (-0.81 to 0.14) | 0.1711 | -0,27 | (-0.73 to 0.20) | 0.2620 |
|  |  |  |  |  |  |  |  |  |
| ***A. lumbricoides*** | |  |  |  |  |  |  |  |
| **neg.*** | 173 | 2,77 | 0,00 | - | - | 0,00 | - | - |
| **pos.** | 47 | 3,59 | 0,78 | (0.23 to 1.33) | 0.0058 | 0,90 | (0.34 to 1.46) | 0.0016 |
|  |  |  |  |  |  |  |  |  |
| ***T. trichiura*** |  |  |  |  |  |  |  |  |
| **neg.*** | 183 | 2,93 | 0,00 | - | - | 0,00 | - | - |
| **pos.** | 37 | 3,04 | -0,27 | (-0.96 to 0.43) | 0.4508 | -0,07 | (-0.70 to 0.57) | 0.8372 |
|  |  |  |  |  |  |  |  |  |
| ***S. mansoni*** |  |  |  |  |  |  |  |  |
| **neg.*** | 141 | 2,86 | 0,00 | - | - | 0,00 | - | - |
| **pos.** | 79 | 3,10 | 0,21 | (-0.26 to 0.69) | 0.3798 | 0,46 | (-0.05 to 0.96) | 0.0769 |
|  |  |  |  |  |  |  |  |  |
| ***S. haematobium*** | |  |  |  |  |  |  |  |
| **neg.*** | 203 | 2,95 | 0,00 | - | - | 0,00 | - | - |
| **pos.** | 17 | 2,93 | -0,02 | (-0.87 to 0.83) | 0.9634 | 0,13 | (-0.70 to 0.96) | 0.7558 |
| *N = number of observations; Mean = mean outcome; Coef. = coefficient; 95% CI = 95% confidence interval* | | | | | | | |  |
| ** reference stratum* | |  |  |  |  |  |  |  |
