# Supplementary material for: Wuchereria bancrofti infection is linked to systemic activation of CD4 and CD8 T cells
Source: PLoS Negl Trop Dis. 2019 Aug 19;13(8):e0007623. doi: 10.1371/journal.pntd.0007623 (PMC6736309; doi:10.1371/journal.pntd.0007623)
Supplement: S2 Table — Uni- and multi-variable mixed-effects linear regression results, with random effect for residence in Kyela site, multivariable models additionally adjusted for age, gender and fever during last 24 hours and different helminth infections. (DOCX) [file pntd.0007623.s003.docx]

**S2 Table:** Association of various factors with percent of HLA-DR^pos^ cells of all CD8 T cells

|  |  |  | **univariable** | | | **multivariable** | | |
| --- | --- | --- | --- | --- | --- | --- | --- | --- |
| **Covariate** | **N** | **Mean** | **Coef.** | **95% CI** | **p-value** | **Coef.** | **95% CI** | **p-value** |
|  |  |  |  |  |  |  |  |  |
| **Age** |  |  |  |  |  |  |  |  |
| **(per year)** | - | - | 0,37 | (0.25 to 0.50) | 0.0000 | 0,38 | (0.25 to 0.51) | 0.0000 |
|  |  |  |  |  |  |  |  |  |
| **Sex** |  |  |  |  |  |  |  |  |
| **female*** | 130 | 22,0 | 0,00 | - | - | 0,00 | - | - |
| **male** | 95 | 22,2 | -1,50 | (-4.60 to 1.60) | 0.3426 | 0,20 | (-2.66 to 3.07) | 0.8886 |
|  |  |  |  |  |  |  |  |  |
| **Current fever** |  |  |  |  |  |  |  |  |
| **no*** | 199 | 21,9 | 0,00 | - | - | 0,00 | - | - |
| **yes** | 20 | 26,4 | 5,29 | (-0.00 to 10.59) | 0.0501 | 3,76 | (-1.09 to 8.61) | 0.1288 |
| **no data** | 6 | 14,0 | -3,24 | (-12.68 to 6.19) | 0.5002 | -2,06 | (-10.70 to 6.58) | 0.6401 |
|  |  |  |  |  |  |  |  |  |
| ***W. bancrofti*** |  |  |  |  |  |  |  |  |
| **neg.*** | 192 | 20,8 | 0,00 | - | - | 0,00 | - | - |
| **pos.** | 33 | 29,5 | 1,69 | (-3.18 to 6.56) | 0.4960 | 0,47 | (-3.96 to 4.89) | 0.8363 |
|  |  |  |  |  |  |  |  |  |
| **Hookworm** |  |  |  |  |  |  |  |  |
| **neg.*** | 146 | 22,7 | 0,00 | - | - | 0,00 | - | - |
| **pos.** | 79 | 21,0 | -2,16 | (-5.34 to 1.01) | 0.1812 | -2,69 | (-5.60 to 0.22) | 0.0698 |
|  |  |  |  |  |  |  |  |  |
| ***A. lumbricoides*** | |  |  |  |  |  |  |  |
| **neg.*** | 176 | 20,8 | 0,00 | - | - | 0,00 | - | - |
| **pos.** | 49 | 26,8 | 3,15 | (-0.61 to 6.90) | 0.1002 | 3,42 | (-0.19 to 7.04) | 0.0634 |
|  |  |  |  |  |  |  |  |  |
| ***T. trichiura*** |  |  |  |  |  |  |  |  |
| **neg.*** | 188 | 20,0 | 0,00 | - | - | 0,00 | - | - |
| **pos.** | 37 | 32,7 | 6,74 | (1.99 to 11.49) | 0.0054 | 6,66 | (2.12 to 11.19) | 0.0040 |
|  |  |  |  |  |  |  |  |  |
| ***S. mansoni*** |  |  |  |  |  |  |  |  |
| **neg.*** | 145 | 22,8 | 0,00 | - | - | 0,00 | - | - |
| **pos.** | 80 | 20,7 | -2,81 | (-5.97 to 0.35) | 0.0810 | 1,06 | (-2.15 to 4.28) | 0.5161 |
|  |  |  |  |  |  |  |  |  |
| ***S. haematobium*** | |  |  |  |  |  |  |  |
| **neg.*** | 208 | 22,2 | 0,00 | - | - | 0,00 | - | - |
| **pos.** | 17 | 21,1 | -1,17 | (-6.92 to 4.57) | 0.6890 | 1,79 | (-3.47 to 7.06) | 0.5050 |
| *N = number of observations; Mean = mean outcome; Coef. = coefficient; 95% CI = 95% confidence interval* | | | | | | | |  |
| ** reference stratum* | |  |  |  |  |  |  |  |
