# Supplementary material for: Wuchereria bancrofti infection is linked to systemic activation of CD4 and CD8 T cells
Source: PLoS Negl Trop Dis. 2019 Aug 19;13(8):e0007623. doi: 10.1371/journal.pntd.0007623 (PMC6736309; doi:10.1371/journal.pntd.0007623)
Supplement: S3 Table — Uni- and multi-variable mixed-effects linear regression results, with random effect for residence in Kyela site, multivariable models additionally adjusted for age, gender and fever during last 24 hours and different helminth infections. (DOCX) [file pntd.0007623.s004.docx]

**S3 Table:** Association of various factors with percent of HLA-DR^pos^CD38^pos^ cells of all CD8 T cells

|  |  |  | **univariable** | | | **multivariable** | | |
| --- | --- | --- | --- | --- | --- | --- | --- | --- |
| **Covariate** | **N** | **Mean** | **Coef.** | **95% CI** | **p-value** | **Coef.** | **95% CI** | **p-value** |
|  |  |  |  |  |  |  |  |  |
| **Age** |  |  |  |  |  |  |  |  |
| **(per year)** | - | - | 0,07 | (-0.00 to 0.14) | 0.0621 | 0,06 | (-0.01 to 0.14) | 0.0996 |
|  |  |  |  |  |  |  |  |  |
| **Sex** |  |  |  |  |  |  |  |  |
| **female*** | 128 | 8,23 | 0,00 | - | - | 0,00 | - | - |
| **male** | 93 | 8,92 | 0,04 | (-1.62 to 1.69) | 0.9656 | 0,66 | (-1.00 to 2.31) | 0.4358 |
|  |  |  |  |  |  |  |  |  |
| **Current fever** |  |  |  |  |  |  |  |  |
| **no*** | 195 | 8,45 | 0,00 | - | - | 0,00 | - | - |
| **yes** | 20 | 10,07 | 1,95 | (-0.87 to 4.77) | 0.1749 | 1,63 | (-1.14 to 4.40) | 0.2480 |
| **no data** | 6 | 5,53 | -0,96 | (-5.98 to 4.05) | 0.7062 | -1,17 | (-6.10 to 3.75) | 0.6402 |
|  |  |  |  |  |  |  |  |  |
| ***W. bancrofti*** |  |  |  |  |  |  |  |  |
| **neg.*** | 189 | 7,69 | 0,00 | - | - | 0,00 | - | - |
| **pos.** | 32 | 13,42 | 3,49 | (0.94 to 6.04) | 0.0072 | 3,45 | (0.91 to 5.98) | 0.0077 |
|  |  |  |  |  |  |  |  |  |
| **Hookworm** |  |  |  |  |  |  |  |  |
| **neg.*** | 143 | 8,69 | 0,00 | - | - | 0,00 | - | - |
| **pos.** | 78 | 8,22 | -0,62 | (-2.31 to 1.08) | 0.4762 | -0,61 | (-2.28 to 1.06) | 0.4736 |
|  |  |  |  |  |  |  |  |  |
| ***A. lumbricoides*** | |  |  |  |  |  |  |  |
| **neg.*** | 173 | 7,96 | 0,00 | - | - | 0,00 | - | - |
| **pos.** | 48 | 10,55 | 1,47 | (-0.54 to 3.47) | 0.1511 | 1,85 | (-0.22 to 3.92) | 0.0801 |
|  |  |  |  |  |  |  |  |  |
| ***T. trichiura*** |  |  |  |  |  |  |  |  |
| **neg.*** | 184 | 8,06 | 0,00 | - | - | 0,00 | - | - |
| **pos.** | 37 | 10,80 | -0,87 | (-3.43 to 1.69) | 0.5040 | -0,79 | (-3.37 to 1.79) | 0.5494 |
|  |  |  |  |  |  |  |  |  |
| ***S. mansoni*** |  |  |  |  |  |  |  |  |
| **neg.*** | 143 | 8,39 | 0,00 | - | - | 0,00 | - | - |
| **pos.** | 78 | 8,75 | 0,03 | (-1.67 to 1.73) | 0.9744 | 0,52 | (-1.33 to 2.38) | 0.5818 |
|  |  |  |  |  |  |  |  |  |
| ***S. haematobium*** | |  |  |  |  |  |  |  |
| **neg.*** | 206 | 8,58 | 0,00 | - | - | 0,00 | - | - |
| **pos.** | 15 | 7,72 | -1,17 | (-4.39 to 2.05) | 0.4747 | -0,78 | (-3.97 to 2.41) | 0.6337 |
| *N = number of observations; Mean = mean outcome; Coef. = coefficient; 95% CI = 95% confidence interval* | | | | | | | |  |
| ** reference stratum* | |  |  |  |  |  |  |  |
