# Supplementary material for: Wuchereria bancrofti infection is linked to systemic activation of CD4 and CD8 T cells
Source: PLoS Negl Trop Dis. 2019 Aug 19;13(8):e0007623. doi: 10.1371/journal.pntd.0007623 (PMC6736309; doi:10.1371/journal.pntd.0007623)
Supplement: S4 Table — Uni- and multi-variable mixed-effects linear regression results, with random effect for residence in Kyela site, multivariable models additionally adjusted for age, gender and fever during last 24 hours and different helminth infections. (DOCX) [file pntd.0007623.s005.docx]

**S4 Table:** Association of various factors with percent of CD27^neg^CD45RO^pos^ cells of all CD4 T cells

|  |  |  | **univariable** | | | **multivariable** | | |
| --- | --- | --- | --- | --- | --- | --- | --- | --- |
| **Covariate** | **N** | **Mean** | **Coef.** | **95% CI** | **p-value** | **Coef.** | **95% CI** | **p-value** |
|  |  |  |  |  |  |  |  |  |
| **Age** |  |  |  |  |  |  |  |  |
| **(per year)** | - | - | 0,25 | (0.16 to 0.34) | 0.0000 | 0,24 | (0.15 to 0.33) | 0.0000 |
|  |  |  |  |  |  |  |  |  |
| **Sex** |  |  |  |  |  |  |  |  |
| **female*** | 126 | 20,75 | 0,00 | - | - | 0,00 | - | - |
| **male** | 94 | 20,44 | -0,56 | (-2.75 to 1.63) | 0.6147 | 0,58 | (-1.48 to 2.65) | 0.5793 |
|  |  |  |  |  |  |  |  |  |
| **Current fever** |  |  |  |  |  |  |  |  |
| **no*** | 194 | 20,70 | 0,00 | - | - | 0,00 | - | - |
| **yes** | 20 | 21,62 | 1,02 | (-2.71 to 4.74) | 0.5933 | 0,06 | (-3.43 to 3.54) | 0.9752 |
| **no data** | 6 | 14,60 | -5,52 | (-12.13 to 1.09) | 0.1019 | -5,43 | (-11.59 to 0.73) | 0.0841 |
|  |  |  |  |  |  |  |  |  |
| ***W. bancrofti*** |  |  |  |  |  |  |  |  |
| **neg.*** | 189 | 20,04 | 0,00 | - | - | 0,00 | - | - |
| **pos.** | 31 | 24,10 | 4,06 | (0.99 to 7.13) | 0.0096 | 3,16 | (0.17 to 6.15) | 0.0383 |
|  |  |  |  |  |  |  |  |  |
| **Hookworm** |  |  |  |  |  |  |  |  |
| **neg.*** | 144 | 20,99 | 0,00 | - | - | 0,00 | - | - |
| **pos.** | 76 | 19,90 | -1,15 | (-3.40 to 1.11) | 0.3195 | -1,59 | (-3.71 to 0.52) | 0.1397 |
|  |  |  |  |  |  |  |  |  |
| ***A. lumbricoides*** | |  |  |  |  |  |  |  |
| **neg.*** | 173 | 20,08 | 0,00 | - | - | 0,00 | - | - |
| **pos.** | 47 | 22,58 | 2,21 | (-0.43 to 4.86) | 0.1007 | 2,48 | (-0.07 to 5.03) | 0.0566 |
|  |  |  |  |  |  |  |  |  |
| ***T. trichiura*** |  |  |  |  |  |  |  |  |
| **neg.*** | 183 | 20,23 | 0,00 | - | - | 0,00 | - | - |
| **pos.** | 37 | 22,52 | 1,99 | (-0.99 to 4.98) | 0.1899 | 1,58 | (-1.30 to 4.46) | 0.2831 |
|  |  |  |  |  |  |  |  |  |
| ***S. mansoni*** |  |  |  |  |  |  |  |  |
| **neg.*** | 141 | 21,11 | 0,00 | - | - | 0,00 | - | - |
| **pos.** | 79 | 19,73 | -1,51 | (-3.74 to 0.73) | 0.1860 | 0,56 | (-1.74 to 2.86) | 0.6334 |
|  |  |  |  |  |  |  |  |  |
| ***S. haematobium*** | |  |  |  |  |  |  |  |
| **neg.*** | 203 | 20,73 | 0,00 | - | - | 0,00 | - | - |
| **pos.** | 17 | 19,23 | -1,52 | (-5.55 to 2.50) | 0.4578 | -0,10 | (-3.87 to 3.67) | 0.9585 |
| *N = number of observations; Mean = mean outcome; Coef. = coefficient; 95% CI = 95% confidence interval* | | | | | | | |  |
| ** reference stratum* | |  |  |  |  |  |  |  |
