# Supplementary material for: Wuchereria bancrofti infection is linked to systemic activation of CD4 and CD8 T cells
Source: PLoS Negl Trop Dis. 2019 Aug 19;13(8):e0007623. doi: 10.1371/journal.pntd.0007623 (PMC6736309; doi:10.1371/journal.pntd.0007623)
Supplement: S5 Table — Uni- and multi-variable mixed-effects linear regression results, with random effect for residence in Kyela site, multivariable models additionally adjusted for age, gender and fever during last 24 hours and different helminth infections. (DOCX) [file pntd.0007623.s006.docx]

**S5 Table:** Association of various factors with percent of CD25^high^FOXP3^pos^ cells of all CD4 T cells

|  |  |  | **univariable** | | | **multivariable** | | |
| --- | --- | --- | --- | --- | --- | --- | --- | --- |
| **Covariate** | **N** | **Mean** | **Coef.** | **95% CI** | **p-value** | **Coef.** | **95% CI** | **p-value** |
|  |  |  |  |  |  |  |  |  |
| **Age** |  |  |  |  |  |  |  |  |
| **(per year)** | - | - | 0,01 | (-0.00 to 0.02) | 0.1823 | 0,01 | (-0.00 to 0.03) | 0.0651 |
|  |  |  |  |  |  |  |  |  |
| **Sex** |  |  |  |  |  |  |  |  |
| **female*** | 118 | 2,189 | 0,00 | - | - | 0,00 | - | - |
| **male** | 90 | 2,414 | 0,21 | (-0.09 to 0.50) | 0.1687 | 0,28 | (-0.02 to 0.58) | 0.0696 |
|  |  |  |  |  |  |  |  |  |
| **Current fever** |  |  |  |  |  |  |  |  |
| **no*** | 183 | 2,291 | 0,00 | - | - | 0,00 | - | - |
| **yes** | 20 | 2,320 | 0,04 | (-0.46 to 0.53) | 0.8839 | -0,02 | (-0.51 to 0.47) | 0.9345 |
| **no data** | 5 | 2,000 | -0,23 | (-1.18 to 0.73) | 0.6384 | -0,11 | (-1.05 to 0.84) | 0.8259 |
|  |  |  |  |  |  |  |  |  |
| ***W. bancrofti*** |  |  |  |  |  |  |  |  |
| **neg.*** | 178 | 2,258 | 0,00 | - | - | 0,00 | - | - |
| **pos.** | 30 | 2,453 | 0,13 | (-0.30 to 0.57) | 0.5491 | 0,03 | (-0.40 to 0.45) | 0.8983 |
|  |  |  |  |  |  |  |  |  |
| **Hookworm** |  |  |  |  |  |  |  |  |
| **neg.*** | 133 | 2,327 | 0,00 | - | - | 0,00 | - | - |
| **pos.** | 75 | 2,215 | -0,12 | (-0.42 to 0.19) | 0.4507 | -0,19 | (-0.50 to 0.11) | 0.2187 |
|  |  |  |  |  |  |  |  |  |
| ***A. lumbricoides*** | |  |  |  |  |  |  |  |
| **neg.*** | 158 | 2,250 | 0,00 | - | - | 0,00 | - | - |
| **pos.** | 50 | 2,402 | 0,12 | (-0.22 to 0.46) | 0.4904 | 0,23 | (-0.13 to 0.58) | 0.2113 |
|  |  |  |  |  |  |  |  |  |
| ***T. trichiura*** |  |  |  |  |  |  |  |  |
| **neg.*** | 171 | 2,216 | 0,00 | - | - | 0,00 | - | - |
| **pos.** | 37 | 2,611 | 0,39 | (0.02 to 0.77) | 0.0414 | 0,40 | (-0.00 to 0.80) | 0.0529 |
|  |  |  |  |  |  |  |  |  |
| ***S. mansoni*** |  |  |  |  |  |  |  |  |
| **neg.*** | 137 | 2,272 | 0,00 | - | - | 0,00 | - | - |
| **pos.** | 71 | 2,314 | 0,04 | (-0.26 to 0.35) | 0.7840 | 0,20 | (-0.14 to 0.54) | 0.2473 |
|  |  |  |  |  |  |  |  |  |
| ***S. haematobium*** | |  |  |  |  |  |  |  |
| **neg.*** | 190 | 2,306 | 0,00 | - | - | 0,00 | - | - |
| **pos.** | 18 | 2,078 | -0,23 | (-0.75 to 0.29) | 0.3845 | -0,10 | (-0.62 to 0.42) | 0.6984 |
| *N = number of observations; Mean = mean outcome; Coef. = coefficient; 95% CI = 95% confidence interval* | | | | | | | |  |
| ** reference stratum* | |  |  |  |  |  |  |  |
