# Supplementary material for: Wuchereria bancrofti infection is linked to systemic activation of CD4 and CD8 T cells
Source: PLoS Negl Trop Dis. 2019 Aug 19;13(8):e0007623. doi: 10.1371/journal.pntd.0007623 (PMC6736309; doi:10.1371/journal.pntd.0007623)
Supplement: S6 Table — Uni- and multi-variable mixed-effects linear regression results, with random effect for residence in Kyela site, multivariable models additionally adjusted for age, gender and fever during last 24 hours and different helminth infections. (DOCX) [file pntd.0007623.s007.docx]

**S6 Table:** Association of various factors with percent of CCR5^pos^ cells of all CD4 T cells

|  |  |  | **univariable** | | | **multivariable** | | |
| --- | --- | --- | --- | --- | --- | --- | --- | --- |
| **Covariate** | **N** | **Mean** | **Coef.** | **95% CI** | **p-value** | **Coef.** | **95% CI** | **p-value** |
|  |  |  |  |  |  |  |  |  |
| **Age** |  |  |  |  |  |  |  |  |
| **(per year)** | - | - | 0,08 | (-0.03 to 0.18) | 0.1549 | 0,06 | (-0.06 to 0.17) | 0.3268 |
|  |  |  |  |  |  |  |  |  |
| **Sex** |  |  |  |  |  |  |  |  |
| **female*** | 113 | 25,57 | 0,00 | - | - | 0,00 | - | - |
| **male** | 87 | 22,47 | -3,10 | (-5.50 to -0.70) | 0.0113 | -2,84 | (-5.33 to -0.35) | 0.0256 |
|  |  |  |  |  |  |  |  |  |
| **Current fever** |  |  |  |  |  |  |  |  |
| **no*** | 175 | 24,13 | 0,00 | - | - | 0,00 | - | - |
| **yes** | 20 | 24,79 | 0,66 | (-3.37 to 4.70) | 0.7466 | 0,19 | (-3.81 to 4.19) | 0.9266 |
| **no data** | 5 | 25,30 | 1,17 | (-6.58 to 8.93) | 0.7664 | 0,32 | (-7.39 to 8.02) | 0.9354 |
|  |  |  |  |  |  |  |  |  |
| ***W. bancrofti*** |  |  |  |  |  |  |  |  |
| **neg.*** | 170 | 24,06 | 0,00 | - | - | 0,00 | - | - |
| **pos.** | 30 | 25,15 | 1,09 | (-2.29 to 4.47) | 0.5280 | 1,45 | (-2.03 to 4.93) | 0.4142 |
|  |  |  |  |  |  |  |  |  |
| **Hookworm** |  |  |  |  |  |  |  |  |
| **neg.*** | 128 | 24,24 | 0,00 | - | - | 0,00 | - | - |
| **pos.** | 72 | 24,18 | -0,06 | (-2.58 to 2.46) | 0.9635 | 0,35 | (-2.17 to 2.88) | 0.7833 |
|  |  |  |  |  |  |  |  |  |
| ***A. lumbricoides*** | |  |  |  |  |  |  |  |
| **neg.*** | 153 | 23,90 | 0,00 | - | - | 0,00 | - | - |
| **pos.** | 47 | 25,27 | 1,37 | (-1.48 to 4.21) | 0.3457 | 1,23 | (-1.73 to 4.19) | 0.4151 |
|  |  |  |  |  |  |  |  |  |
| ***T. trichiura*** |  |  |  |  |  |  |  |  |
| **neg.*** | 164 | 24,42 | 0,00 | - | - | 0,00 | - | - |
| **pos.** | 36 | 23,33 | -1,09 | (-4.24 to 2.05) | 0.4956 | -0,99 | (-4.35 to 2.36) | 0.5615 |
|  |  |  |  |  |  |  |  |  |
| ***S. mansoni*** |  |  |  |  |  |  |  |  |
| **neg.*** | 130 | 24,27 | 0,00 | - | - | 0,00 | - | - |
| **pos.** | 70 | 24,12 | -0,15 | (-2.68 to 2.39) | 0.9084 | 0,53 | (-2.24 to 3.30) | 0.7056 |
|  |  |  |  |  |  |  |  |  |
| ***S. haematobium*** | |  |  |  |  |  |  |  |
| **neg.*** | 182 | 24,33 | 0,00 | - | - | 0,00 | - | - |
| **pos.** | 18 | 23,08 | -1,26 | (-5.48 to 2.96) | 0.5596 | -0,85 | (-5.08 to 3.39) | 0.6957 |
| *N = number of observations; Mean = mean outcome; Coef. = coefficient; 95% CI = 95% confidence interval* | | | | | | | |  |
| ** reference stratum* | |  |  |  |  |  |  |  |
