# Supplementary material for: Wuchereria bancrofti infection is linked to systemic activation of CD4 and CD8 T cells
Source: PLoS Negl Trop Dis. 2019 Aug 19;13(8):e0007623. doi: 10.1371/journal.pntd.0007623 (PMC6736309; doi:10.1371/journal.pntd.0007623)
Supplement: S7 Table — Uni- and multi-variable mixed-effects linear regression results, with random effect for residence in Kyela site, multivariable models additionally adjusted for age, gender and fever during last 24 hours and different helminth infections. (DOCX) [file pntd.0007623.s008.docx]

**S7 Table:** Association of various factors with percentage of CCR5^pos^ of all regulatory CD4 T cells

|  |  |  | **univariable** | | | **multivariable** | | |
| --- | --- | --- | --- | --- | --- | --- | --- | --- |
| **Covariate** | **N** | **Mean** | **Coef.** | **95% CI** | **p-value** | **Coef.** | **95% CI** | **p-value** |
|  |  |  |  |  |  |  |  |  |
| **Age** |  |  |  |  |  |  |  |  |
| **(per year)** | - | - | -0,01 | (-0.18 to 0.15) | 0.8694 | 0,02 | (-0.16 to 0.19) | 0.8608 |
|  |  |  |  |  |  |  |  |  |
| **Sex** |  |  |  |  |  |  |  |  |
| **female*** | 113 | 55,92 | 0,00 | - | - | 0,00 | - | - |
| **male** | 87 | 53,04 | -2,88 | (-6.70 to 0.94) | 0.1391 | -2,24 | (-6.14 to 1.66) | 0.2597 |
|  |  |  |  |  |  |  |  |  |
| **Current fever** |  |  |  |  |  |  |  |  |
| **no*** | 175 | 54,52 | 0,00 | - | - | 0,00 | - | - |
| **yes** | 20 | 56,44 | 1,92 | (-4.42 to 8.26) | 0.5530 | 1,04 | (-5.23 to 7.30) | 0.7459 |
| **no data** | 5 | 52,66 | -1,86 | (-14.05 to 10.32) | 0.7647 | -1,76 | (-13.82 to 10.30) | 0.7747 |
|  |  |  |  |  |  |  |  |  |
| ***W. bancrofti*** |  |  |  |  |  |  |  |  |
| **neg.*** | 170 | 54,47 | 0,00 | - | - | 0,00 | - | - |
| **pos.** | 30 | 55,80 | 1,34 | (-3.99 to 6.66) | 0.6223 | 2,55 | (-2.89 to 8.00) | 0.3585 |
|  |  |  |  |  |  |  |  |  |
| **Hookworm** |  |  |  |  |  |  |  |  |
| **neg.*** | 128 | 55,87 | 0,00 | - | - | 0,00 | - | - |
| **pos.** | 72 | 52,52 | -3,36 | (-7.29 to 0.58) | 0.0946 | -2,52 | (-6.48 to 1.44) | 0.2118 |
|  |  |  |  |  |  |  |  |  |
| ***A. lumbricoides*** | |  |  |  |  |  |  |  |
| **neg.*** | 153 | 54,27 | 0,00 | - | - | 0,00 | - | - |
| **pos.** | 47 | 55,95 | 1,67 | (-2.81 to 6.15) | 0.4638 | 2,58 | (-2.05 to 7.20) | 0.2752 |
|  |  |  |  |  |  |  |  |  |
| ***T. trichiura*** |  |  |  |  |  |  |  |  |
| **neg.*** | 164 | 55,41 | 0,00 | - | - | 0,00 | - | - |
| **pos.** | 36 | 51,27 | -4,14 | (-9.06 to 0.77) | 0.0987 | -3,52 | (-8.77 to 1.73) | 0.1893 |
|  |  |  |  |  |  |  |  |  |
| ***S. mansoni*** |  |  |  |  |  |  |  |  |
| **neg.*** | 130 | 53,61 | 0,00 | - | - | 0,00 | - | - |
| **pos.** | 70 | 56,63 | 3,03 | (-0.94 to 6.99) | 0.1347 | 3,28 | (-1.06 to 7.61) | 0.1385 |
|  |  |  |  |  |  |  |  |  |
| ***S. haematobium*** | |  |  |  |  |  |  |  |
| **neg.*** | 182 | 54,35 | 0,00 | - | - | 0,00 | - | - |
| **pos.** | 18 | 57,91 | 3,56 | (-3.07 to 10.19) | 0.2924 | 3,51 | (-3.12 to 10.15) | 0.2991 |
| *N = number of observations; Mean = mean outcome; Coef. = coefficient; 95% CI = 95% confidence interval* | | | | | | | |  |
| ** reference stratum* | |  |  |  |  |  |  |  |
