# Supplementary material for: Wuchereria bancrofti infection is linked to systemic activation of CD4 and CD8 T cells
Source: PLoS Negl Trop Dis. 2019 Aug 19;13(8):e0007623. doi: 10.1371/journal.pntd.0007623 (PMC6736309; doi:10.1371/journal.pntd.0007623)
Supplement: S8 Table — Uni- and multi-variable mixed-effects linear regression results, with random effect for residence in Kyela site, multivariable models additionally adjusted for age, gender and fever during last 24 hours and different helminth infections. (DOCX) [file pntd.0007623.s009.docx]

**S8 Table:** Association of various factors with mean fluorescence intensity of CCR5 on memory CD4 T cells

|  |  |  | **univariable** | | | **multivariable** | | |
| --- | --- | --- | --- | --- | --- | --- | --- | --- |
| **Covariate** | **N** | **Mean** | **Coef.** | **95% CI** | **p-value** | **Coef.** | **95% CI** | **p-value** |
|  |  |  |  |  |  |  |  |  |
| **Age** |  |  |  |  |  |  |  |  |
| **(per year)** | - | - | -2,84 | (-7.19 to 1.52) | 0.2017 | -3,16 | (-7.74 to 1.42) | 0.1763 |
|  |  |  |  |  |  |  |  |  |
| **Sex** |  |  |  |  |  |  |  |  |
| **female*** | 123 | 788 | 0,00 | - | - | 0,00 | - | - |
| **male** | 93 | 780 | -6,84 | (-109.68 to 96.00) | 0.8963 | -9,49 | (-113.21 to 94.23) | 0.8577 |
|  |  |  |  |  |  |  |  |  |
| **Current fever** |  |  |  |  |  |  |  |  |
| **no*** | 190 | 775 | 0,00 | - | - | 0,00 | - | - |
| **yes** | 20 | 933 | 157,11 | (-16.63 to 330.85) | 0.0763 | 164,65 | (-9.26 to 338.57) | 0.0635 |
| **no data** | 6 | 607 | -176,87 | (-484.04 to 130.30) | 0.2591 | -197,92 | (-505.21 to 109.38) | 0.2068 |
|  |  |  |  |  |  |  |  |  |
| ***W. bancrofti*** |  |  |  |  |  |  |  |  |
| **neg.*** | 185 | 799 | 0,00 | - | - | 0,00 | - | - |
| **pos.** | 31 | 699 | -100,43 | (-244.95 to 44.10) | 0.1732 | -65,07 | (-214.22 to 84.09) | 0.3926 |
|  |  |  |  |  |  |  |  |  |
| **Hookworm** |  |  |  |  |  |  |  |  |
| **neg.*** | 141 | 801 | 0,00 | - | - | 0,00 | - | - |
| **pos.** | 75 | 754 | -46,27 | (-152.91 to 60.38) | 0.3951 | -27,64 | (-134.18 to 78.89) | 0.6111 |
|  |  |  |  |  |  |  |  |  |
| ***A. lumbricoides*** | |  |  |  |  |  |  |  |
| **neg.*** | 171 | 797 | 0,00 | - | - | 0,00 | - | - |
| **pos.** | 45 | 738 | -59,02 | (-184.08 to 66.04) | 0.3550 | -75,25 | (-204.94 to 54.45) | 0.2555 |
|  |  |  |  |  |  |  |  |  |
| ***T. trichiura*** |  |  |  |  |  |  |  |  |
| **neg.*** | 179 | 800 | 0,00 | - | - | 0,00 | - | - |
| **pos.** | 37 | 712 | -87,75 | (-222.31 to 46.81) | 0.2012 | -73,03 | (-216.97 to 70.90) | 0.3200 |
|  |  |  |  |  |  |  |  |  |
| ***S. mansoni*** |  |  |  |  |  |  |  |  |
| **neg.*** | 139 | 776 | 0,00 | - | - | 0,00 | - | - |
| **pos.** | 77 | 801 | 25,72 | (-80.39 to 131.84) | 0.6347 | -26,55 | (-142.53 to 89.44) | 0.6537 |
|  |  |  |  |  |  |  |  |  |
| ***S. haematobium*** | |  |  |  |  |  |  |  |
| **neg.*** | 199 | 781 | 0,00 | - | - | 0,00 | - | - |
| **pos.** | 17 | 827 | 46,51 | (-142.22 to 235.25) | 0.6291 | 13,61 | (-174.54 to 201.76) | 0.8873 |
| *N = number of observations; Mean = mean outcome; Coef. = coefficient; 95% CI = 95% confidence interval* | | | | | | | |  |
| ** reference stratum* | |  |  |  |  |  |  |  |
